# Supplementary material for: Competing correlated states and abundant orbital magnetism in twisted monolayer-bilayer graphene
Source: Nat Commun. 2021 Aug 5;12:4727. doi: 10.1038/s41467-021-25044-1 (PMC8342414; doi:10.1038/s41467-021-25044-1)
Supplement: Supplementary file 1 — Supplementary Information [file 41467_2021_25044_MOESM1_ESM.pdf]

Supplementary Information for: Competing ground states and abundant orbital magnetism in twisted monolayer-bilayer graphene

Minhao He[1], Ya-Hui Zhang[2], Yuhao Li[1], Zaiyao Fei[1], K. Watanabe[3], T. Taniguchi[4], Xiaodong Xu[1,5†], and Matthew Yankowitz[1,5†]
[1] *Department of Physics, University of Washington, Seattle, Washington, 98195, USA*
[2] *Department of Physics, Harvard University, Cambridge, MA, USA*
[2] *National Institute for Materials Science, 1-1 Namiki, Tsukuba 305-0044, Japan*
[3] *Research Center for Functional Materials, National Institute for Materials Science, 1-1 Namiki, Tsukuba 305-0044, Japan*
[4] *International Center for Materials Nanoarchitectonics, National Institute for Materials Science, 1-1 Namiki, Tsukuba 305-0044, Japan*
[5] *Department of Materials Science and Engineering, University of Washington, Seattle, Washington, 98195, USA and*
† *xuxd@uw.edu (X.X.); myank@uw.edu (M.Y.)*

Supplementary Note 1. Band structure calculation and valley Chern number

We calculate the band structure of tMBG using the standard continuum model. The Hamiltonian is

$$H = H_{MG} + H_{BG} + H_M \tag{1}$$

We have

$$H_{MG} = \sum_{\mathbf{k}} (\tilde{c}_A^\dagger(\mathbf{k}), \tilde{c}_B^\dagger(\mathbf{k})) \begin{pmatrix} -\frac{\delta}{2} & -\frac{\sqrt{3}}{2} t(\tilde{k}_x - i\tilde{k}_y) \\ -\frac{\sqrt{3}}{2} t(\tilde{k}_x + i\tilde{k}_y) & -\frac{\delta}{2} \end{pmatrix} \begin{pmatrix} \tilde{c}_A(\mathbf{k}) \\ \tilde{c}_B(\mathbf{k}) \end{pmatrix} \tag{2}$$

where $\tilde{\mathbf{k}} = R(-\theta/2)\mathbf{k}$, with twist angle θ. $R(\varphi)$ is the transformation matrix for anticlockwise rotation with angle φ.
The Hamiltonian for the bilayer graphene is

$$H_{BG} = \sum_{\mathbf{k}} \Psi^\dagger(\mathbf{k}) \begin{pmatrix} 0 & -\frac{\sqrt{3}}{2} t(\tilde{k}_x - i\tilde{k}_y) & -\frac{\sqrt{3}}{2}\gamma_4(\tilde{k}_x - i\tilde{k}_y) & -\frac{\sqrt{3}}{2}\gamma_3(\tilde{k}_x + i\tilde{k}_y) \\ -\frac{\sqrt{3}}{2} t(\tilde{k}_x + i\tilde{k}_y) & 0 & \gamma_1 & -\frac{\sqrt{3}}{2}\gamma_4(\tilde{k}_x - i\tilde{k}_y) \\ -\frac{\sqrt{3}}{2}\gamma_4(\tilde{k}_x + i\tilde{k}_y) & \gamma_1 & \frac{\delta}{2} & -\frac{\sqrt{3}}{2} t(\tilde{k}_x - i\tilde{k}_y) \\ -\frac{\sqrt{3}}{2}\gamma_3(\tilde{k}_x - i\tilde{k}_y) & -\frac{\sqrt{3}}{2}\gamma_4(\tilde{k}_x + i\tilde{k}_y) & -\frac{\sqrt{3}}{2} t(\tilde{k}_x + i\tilde{k}_y) & \frac{\delta}{2} \end{pmatrix} \Psi(\mathbf{k}) \tag{3}$$

where $\tilde{\mathbf{k}} = R(\theta/2)\mathbf{k}$ and $\Psi^\dagger(\mathbf{k}) = (c_{A_1}^\dagger(\mathbf{k}), c_{B_1}^\dagger(\mathbf{k}), c_{A_2}^\dagger(\mathbf{k}), c_{B_2}^\dagger(\mathbf{k}))$.
Finally the interlayer moiré tunneling term is

$$H_M = \sum_{\mathbf{k}} \sum_{j=0,1,2} (\tilde{c}_A^\dagger(\mathbf{k}), \tilde{c}_B^\dagger(\mathbf{k})) \begin{pmatrix} \alpha t_M & t_M e^{-i\frac{2\pi}{3}j} \\ t_M e^{i\frac{2\pi}{3}j} & \alpha t_M \end{pmatrix} \begin{pmatrix} c_{A_1}(\mathbf{k}+\mathbf{Q}_j) \\ c_{B_1}(\mathbf{k}+\mathbf{Q}_j) \end{pmatrix} + h.c. \tag{4}$$

where $\mathbf{Q}_0 = (0,0)$, $\mathbf{Q}_1 = \frac{1}{a_M}(-\frac{2\pi}{\sqrt{3}}, -2\pi)$ and $\mathbf{Q}_2 = \frac{1}{a_M}(\frac{2\pi}{\sqrt{3}}, -2\pi)$, with a_M as the moiré lattice constant.

We use parameters $(t, \gamma_1, \gamma_3, \gamma_4) = (-2610, 361, 283, 140)$ meV. For the interlayer tunneling, we use $t_M = 110$ meV and $\alpha = 0.5$. δ is the potential difference, which is tuned by the displacement field, D.

The dispersion at $\delta = -40$ meV and $\delta = 40$ meV for twist angle $\theta = 1.16°$ is shown in Fig. 1. The valley Chern numbers, C_v, for the conduction band are 1 and 2, respectively. Note that we take a convention in which $\delta > 0$ corresponds to the electric field pointing from the monolayer to the bilayer graphene (similarly, this corresponds to $D > 0$).

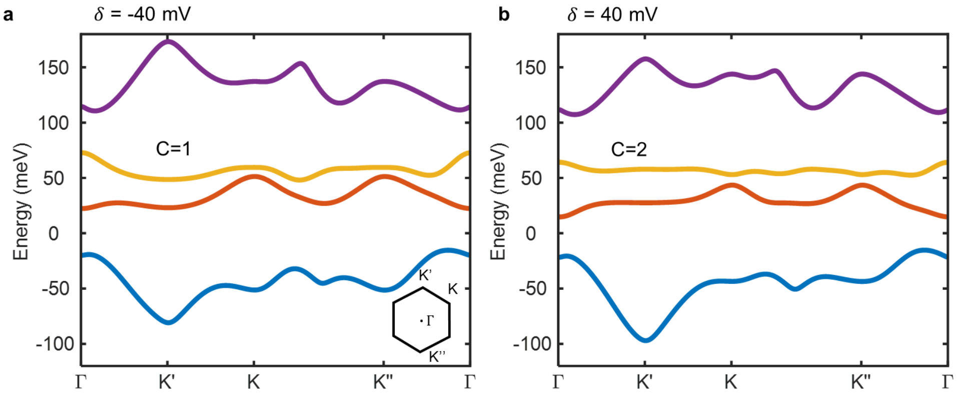

Supplementary Figure 1. **Band structure of tMBG at twist angle** $\theta = 1.16°$**.** **a**, $\delta = -40$ meV and **b**, $\delta = 40$ meV. The valley Chern number of the lowest moiré conduction band (yellow) is $C_v = 1$ for $\delta = -40$ meV, and $C_v = 2$ for $\delta = 40$ meV. The inset in **a** shows the relevant high symmetry points in the first Brillouin zone.

Supplementary Note 2. Calculation of valley wave dispersion

For simplicity, we project the Hamiltonian to include only the conduction band. However, the other remote bands still play a role in renormalizing the dispersion of the active conduction band from the interaction:

$$H_V = \frac{1}{2}\frac{1}{N_s}\sum_{\mathbf{q}} V(\mathbf{q}) \sum_{\mathbf{k}_1}\sum_{\mathbf{k}_2} c^\dagger_{a;m_1}(\mathbf{k}_1+\mathbf{q}) c^\dagger_{b;n_1}(\mathbf{k}_2-\mathbf{q}) c_{b;n_2}(\mathbf{k}_2) c_{a;m_2}(\mathbf{k}_1) \lambda_{a;m_1m_2}(\mathbf{k}_1,\mathbf{q})\lambda_{b;n_1n_2}(\mathbf{k}_2,-\mathbf{q}) \tag{5}$$

where,

$$V(\mathbf{q}) = \frac{15\text{nm}}{a_M}\frac{1392\text{meV}}{\epsilon}\frac{1}{qa_M}\tanh(qr_0) \tag{6}$$

Here r_0 is the screening length from the metallic contact, which we use $r_0 = 5a_M$. In the above $\lambda_a; mn(\mathbf{k},\mathbf{q}) = \langle \mu_{a;m}(\mathbf{k}+\mathbf{q})|\mu_{b;n}(\mathbf{k})\rangle$ is the form factor. Here $a, b = +, -$ is the valley index and m, n is the band index.

Let us fully fill all of the bands below the conduction band. Then the kinetic term of the conduction band is renormalized:

$$H_K = H_K^0 + \sum_{\mathbf{k}} \xi_H(\mathbf{k}) c^\dagger(\mathbf{k})c(\mathbf{k}) + \sum_{\mathbf{k}} \xi_F(\mathbf{k}) c^\dagger(\mathbf{k})c(\mathbf{k}) \tag{7}$$

where c is the operator for the valley $+$ of the conduction band. ϵ_H is from the Hartree term and ϵ_F is from the Fock term.

We have

$$\xi_H(\mathbf{k}) = \sum_{\mathbf{G}_M} V(\mathbf{G}_M)\rho(\mathbf{G}_M)\lambda_+(\mathbf{k},\mathbf{G}_M) \tag{8}$$

where,

$$\rho(\mathbf{G}_M) = 4\frac{1}{N_s}\sum_{m\in O}\lambda_{a;mm}(\mathbf{k},\mathbf{G}_M) \tag{9}$$

Here the factor 4 comes from spin and valley degeneracy. O is the set of occupied bands.

The Fock term is

$$\xi_F(\mathbf{k}) = -\frac{1}{N_s}\sum_{\mathbf{q}} V(\mathbf{q}) \sum_{m\in O} |\lambda_{a;mc}(\mathbf{k},\mathbf{q})|^2 \tag{10}$$

where $m \in O$ is the index of the occupied band. c is the index of the conduction band.

Next, we calculate the valley wave assuming that the ground state is spin-valley polarized at $\nu = 1$. Without loss of generality, we assume the ground state fully occupies $+, \downarrow$. A collective excitation with momentum $\mathbf{q}$ is generated by a particle-hole boson $b_{ab;\mathbf{q}}(\mathbf{k})^\dagger = c^\dagger_{a;\uparrow}(\mathbf{k}+\mathbf{q})c_{b;\downarrow}(\mathbf{k})$, where $a, b = +, -$ labels the valley. Due to the $SU(2)\times SU(2)$ symmetry, the dispersion of the exciton corresponding to $c^\dagger_{+;\sigma}(\mathbf{k}+\mathbf{q})c_{-;\sigma'}(\mathbf{k})$ does not depend on the spin index $\sigma\sigma'$. In the following we will only consider the spin-singlet valley-flip exciton.

For a fixed momentum $\mathbf{q}$, we can derive Hamiltonian for $b_{ab;\mathbf{q}}(\mathbf{k})$:

$$H_{ab}(\mathbf{q}) = H^V_{ab}(\mathbf{q}) + H^K_{ab}(\mathbf{q}) \tag{11}$$

where,

$$\begin{aligned} H^V_{ab}(\mathbf{q}) = &-\sum_{\mathbf{k}\in MBZ}\sum_{\tilde{\mathbf{q}}} V(\tilde{\mathbf{q}})\big(\lambda_a(\mathbf{k}+\mathbf{q},\tilde{\mathbf{q}})\lambda_b(\mathbf{k}+\tilde{\mathbf{q}},-\tilde{\mathbf{q}})b^\dagger_{ab;\mathbf{q}}(\mathbf{k}+\tilde{\mathbf{q}})b_{ab;\mathbf{q}}(\mathbf{k}) \\ &+\sum_{\tilde{\mathbf{q}}} V(\tilde{\mathbf{q}})\lambda_b(\mathbf{k},\tilde{\mathbf{q}})\lambda_b(\mathbf{k}+\tilde{\mathbf{q}},-\tilde{\mathbf{q}})b^\dagger_{ab;\mathbf{q}}(\mathbf{k})b_{ab;\mathbf{q}}(\mathbf{k}) \end{aligned} \tag{12}$$

and

$$H^K_{ab}(\mathbf{q}) = \sum_{\mathbf{k}\in MBZ} (\xi_a(\mathbf{k}+\mathbf{q}) - \xi_b(\mathbf{k}))b^\dagger_{ab;\mathbf{q}}(\mathbf{k})b_{ab;\mathbf{q}}(\mathbf{k}) \tag{13}$$

where ξ_a is the dispersion after including the renormalization from remote bands.

For a fixed momentum $\mathbf{q}$, the ground state of $H_{ab}(\mathbf{q})$ gives the energy of the excitation, $\omega_{ab}(\mathbf{q})$. Here $\omega_{++}(\mathbf{q})$ is the spin wave excitation and $\omega_{+-}(\mathbf{q})$ is the spin-valley wave excitation.

We focus on the intervalley exciton $\omega_{+-}(\mathbf{q})$. As shown in Fig. 2c of the main text, when we increase the dielectric constant ϵ, the minimum energy of the exciton becomes negative at momentum $\mathbf{q} = K'$ in the mini Brillouin Zone (MBZ), suggesting an instability of the polarized ground state towards an intervalley coherent (IVC) state. As the magnon dispersion has its minimum at a non-zero momentum, the resulting IVC order must also carry a non-zero momentum, and is distinct from the IVC previously proposed in tBLG [1]. We denote this as a "Q-IVC" to emphasize the non-zero momentum $\mathbf{Q}$.

Supplementary Note 3. Symmetry analysis of Q-IVC order

As discussed above, the valley polarized ground state is unstable because the energy of the valley magnon (or exciton) becomes negative when we increase the dielectric constant ϵ. The condensation of valley magnons will lead to an IVC state. Here we perform a general symmetry analysis of the possible IVC states. Given that the minimum of valley magnon dispersion is generically at non-zero momentum $\mathbf{Q}$, the resulting IVC carries momentum $\mathbf{Q}$.

We consider a general IVC ansatz described by a mean field theory:

$$H_M = -\sum_{\mathbf{k}}[F(\mathbf{k})c^\dagger_+(\mathbf{k}+\frac{1}{2}\mathbf{Q})c_-(\mathbf{k}-\frac{1}{2}\mathbf{Q}) + F^*(\mathbf{k})c^\dagger_-(\mathbf{k}-\frac{1}{2}\mathbf{Q})c_+(\mathbf{k}+\frac{1}{2}\mathbf{Q})] \tag{14}$$

where $F(\mathbf{k})$ represents the profile of the intervalley exciton, similar to $\Delta(\mathbf{k})$ in Cooper pairing. Here we assume that the exciton is condensed at momentum $\mathbf{Q}$, which may not be zero.

Using $Tc_+(\mathbf{k})T^{-1} = c_-(-\mathbf{k})$ and $Tc_-(\mathbf{k})T^{-1} = c_+(-\mathbf{k})$, we find:

$$TH_MT^{-1} = -\sum_{\mathbf{k}}[F^*(\mathbf{k})c^\dagger_-(-\mathbf{k}-\frac{1}{2}\mathbf{Q})c_+(-\mathbf{k}+\frac{1}{2}\mathbf{Q}) + F(\mathbf{k})c^\dagger_+(-\mathbf{k}+\frac{1}{2}\mathbf{Q})c_-(-\mathbf{k}-\frac{1}{2}\mathbf{Q}) \tag{15}$$

Then the time reversal symmetry $H_M = TH_MT^{-1}$ requires

$$F(\mathbf{k}) = F(-\mathbf{k}) \tag{16}$$

For an IVC state in the case of non-zero C_v, $F(\mathbf{k})$ needs to acquire the Berry phase of the two bands with opposite signs of the Chern number. A general argument shows that the complex field $F(\mathbf{k})$ cannot be a constant in momentum space, and should have total vorticity equal to $2C_v$. As a result, one usually finds that $F(\mathbf{k}) \neq F(-\mathbf{k})$. This is reflected in our direct calculation of the intervalley exciton wavefunction on top of the spin-valley polarized ground state (see the inset of Fig. 2c of the main text). As a consequence, the Q-IVC order breaks the time reversal symmetry. Note that there is always a degenerate IVC state with $\tilde{F}(\mathbf{k}) = F(-\mathbf{k})$ as the time reversal partner of the IVC with $F(\mathbf{k})$. These two IVC states are degenerate at zero magnetic field, but split for $|B| > 0$. This degeneracy can also be broken spontaneously at $B = 0$.

Let us also comment on the translation symmetry for the Q-IVC order. If only the IVC corresponding to one momentum $\mathbf{Q}$ is condensed, the resulting phase still preserves the moiré translation symmetry because we can apply a global phase generated by the valley charge. We call the IVC in this class a uniform Q-IVC. Note that the non-zero $\mathbf{Q}$ can be gauged away by applying a constant gauge field $\mathbf{A}\tau_z$, where τ_z is the Pauli matrix in valley space and $\mathbf{A} = -\mathbf{Q}$. Theoretically the Q-IVC order can hence be treated in the same way as the usual IVC with $\mathbf{Q} = 0$, and a gap will be opened if the Q-IVC order is sufficiently strong at integer filling. Microscopically, however, the momentum $\mathbf{Q}$ is still meaningful when considering the density profile at the atomic scale: $\rho(\mathbf{r}) = \Psi^\dagger_K(\mathbf{r})\psi_K(\mathbf{r}) + \Psi^\dagger_{K'}(\mathbf{r})\psi_{K'}(\mathbf{r}) + (\Psi^\dagger_K(\mathbf{r})\psi_{K'}(\mathbf{r})e^{i\mathbf{k}\cdot\mathbf{r}} + h.c.)$, where $\Psi_{K/K'}(\mathbf{r})$ is the electron operator for the valley K and K' respectively. If there is a Q-IVC order, $\langle\langle\psi^\dagger_K(\mathbf{r})\psi_{K'}(\mathbf{r})\rangle \sim e^{i\mathbf{Q}\cdot\mathbf{r}}$, and as a result the density profile will have a component $\rho(\mathbf{r}) = \rho_0 + A\cos((\mathbf{K}+\mathbf{Q})\cdot\mathbf{r})$. Such a density profile with momentum $\mathbf{K}+\mathbf{Q}$ can in principle be observed with scanning tunneling microscopy. Note here that $|\mathbf{Q}| \sim \frac{4\pi}{3a_M}$ where a_M is the moiré lattice constant. On the other hand $|\mathbf{K}| = \frac{4\pi}{3a}$

is much larger given that a is the original graphene lattice constant. Therefore the momentum $\mathbf{K}+\mathbf{Q}$ of the density profile is only slightly different from the Kekulé pattern with momentum $\mathbf{K}$.

However, if the IVC with at least two distinct momenta $\mathbf{Q}_1, \mathbf{Q}_2$ condenses, the final state breaks the moiré translation symmetry and there is a charge density wave order (CDW) at momentum $\mathbf{Q}_1 - \mathbf{Q}_2$. If there is a C_3 symmetry, the valley magnon dispersion should have minima at three $\mathbf{Q}$ related by the C_3 rotation. However, when there is strain, C_3 is broken and one momentum will be selected and the final phase is likely to be the uniform Q-IVC order with only one momentum $\mathbf{Q}$.

Supplementary Note 4. Summary of observed correlated states in tMBG

Table 1 summarizes the correlated states observed at all integer ν in the conduction band of tMBG for both signs of D in devices over a range of twist angles, $0.89^\circ \leq \theta \leq 1.385^\circ$. The data are compiled from devices D1, D2, and D3 reported here, as well as from devices D1 and D2 of Ref. [2] and devices D1, D3, and D4 of Ref. [3]. The rotational alignment of the tMBG to the top and bottom encapsulating BN is a latent parameter. We have no reliable estimate of these twist angles for any of our devices, nor do we see obvious signatures of close rotational alignment, however this may nevertheless play a role in driving variations between tMBG devices with otherwise comparable twist angles.

We now comment on a number of details of the collectively reported tMBG devices. First, we note that in principle it possible that devices with $\theta \geq 1.25^\circ$ from Refs. [3] and [4] also exhibit weak AHE around $\nu = 1$ and 3 for $D < 0$ states, however such measurements are not reported in those manuscripts, so we are unable to make such a determination. We also note that a second device with $\theta = 1.25^\circ$ was also reported in Ref. [3], exhibiting a very similar phase diagram to the first. However, measurements of the AHE are not shown, and we therefore omit it from the table. Finally, we note that devices with larger twist angles up to $\sim 1.6^\circ$ have exhibited signatures of symmetry broken states for $D > 0$ [2, 4], however these states all exhibit metallic temperature dependence and have not been investigated in detail. We also omit these devices from the table.

Although identical sets of measurements have not been performed for all devices, the table appears to reveal a number of trends in the evolution of the correlated states with θ. For $D > 0$, the $\nu = 2$ state is a robust trivial insulator over a wide range of twist angles, and appears to be spin-polarized from measurements of the gap with in-plane magnetic field, $B_{||}$ [2]. A Chern insulator state at $B = 0$ appears to emerge around $\nu = 3$ only for a small range of twist angles near 1.25°. The AHE appears to approach a quantization at $h/2e^2$, suggesting the band has $C_v = 2$ at this twist angle. For other twist angles, symmetry-broken states at $\nu = 3$ are only observed at finite B. The $\nu = 1$ state exhibits more complicated behavior. A gapped state is observed in one device with $\theta = 0.89^\circ$ [2], but not in another at 0.90° [3]. In the former, a large AHE is observed, with quantization that appears to approach h/e^2, suggesting the band has $C_v = 1$ at this twist angle. This behavior is consistent with a theoretically anticipated topological transition with twist angle around $\theta = 1.0^\circ$ [5]. The gap closes as the twist angle increases to around 1.05°, before reemerging again in devices with twist angles larger than 1.13°. The unusual behavior surrounding this state has been discussed at length in the main text, but in short appears to be consistent with a transition from an IVC to VP state with increasing twist angle. The absence of a gapped state at slightly smaller twist angles of $\sim 1.05^\circ$ is superficially consistent with this picture, understood collectively as a decrease in U/W as the twist angle grows over this range of angles.

For $D < 0$, the behavior of the correlated states appears to be less sensitive to twist angle. Correlated states with metallic temperature dependence are observed in devices within the approximate range $1.05^\circ \lesssim \theta \lesssim 1.25^\circ$. The lone exception is the $\nu = 1$ state in device D2, which becomes weakly insulating at temperatures below 1 K. In all devices for which there are suitable measurements, the correlated metallic states at $\nu = 1$ and 3 have an associated AHE. Although overlooked initially, we note that a retrospective analysis of the $\theta = 1.05 - 1.08^\circ$ device from Ref. [2] also reveals a similar AHE to the devices we report here. Gradual transitions to insulating behavior are observed upon increasing B at $\nu = 1$, whereas abrupt transitions (accompanied by hysteresis in device D2) are observed at $\nu = 2$ and 3. Previous measurements from device D1 in Ref. [2] show that these states are nearly insensitive to $B_{||}$, suggesting that all of the phase transitions are driven by orbital effects.

	$D>0$, $\nu=1$	$D>0$, $\nu=2$	$D>0$, $\nu=3$	$D<0$, $\nu=1$	$D<0$, $\nu=2$	$D<0$, $\nu=3$
0.89° (Ref. [2])	Chern insulator (approaching quantization with $C=1$)	weakly insulating	none	none	none	none
0.90° (Ref. [3])	none	none	none	none	none	none
$1.05-1.08°$ (Ref. [2])	none (weak state emerges in large $B_{\vert\vert}$)	trivial insulator (gap grows with $B_{\vert\vert}$, suggesting spin-polarized)	none (weak state emerges in large $B_{\vert\vert}$)	metallic resistive state with AHE (incipient insulator emerges gradually with B)	metallic resistive state (insulator emerges abruptly at finite B)	metallic resistive state with AHE (insulator emerges abruptly at finite B)
1.13° (device D1)	trivial insulator (AHE upon doping)	trivial insulator	none	metallic resistive state with AHE (insulator emerges gradually with B)	metallic resistive state (insulator emerges abruptly at finite B)	metallic resistive state with AHE (insulator emerges abruptly at finite B)
1.14° (device D3)	trivial insulator (AHE upon doping)	trivial insulator	none	metallic resistive state with AHE (insulator emerges gradually with B)	metallic resistive state (insulator emerges abruptly at finite B)	metallic resistive state with AHE (insulator emerges abruptly at finite B)
1.19° (device D2)	trivial insulator (AHE upon doping)	trivial insulator	obscured by poor contacts	trivial insulator (AHE upon doping)	metallic resistive state (insulator emerges abruptly at finite B)	metallic resistive state with AHE (insulator emerges abruptly at finite B)
1.25° (Ref. [3])	Chern insulator (approaching quantization with $C=2$)	trivial insulator	Chern insulator (approaching quantization with $C=2$)	metallic resistive state	metallic resistive state	metallic resistive state
1.385° (Ref. [3])	metallic with weak AHE	trivial insulator	symmetry-broken metal	none	none	none

Supplementary Table 1. Summary of the properties of the correlated states in devices D1, D2, and D3, as well as in selected devices from Refs. [2, 3]. Entries reading “none” indicate that no correlated state is clearly observed or reported.

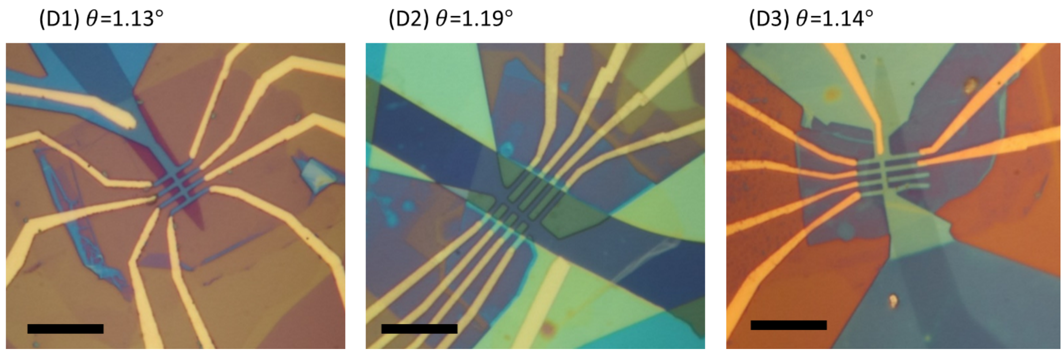

Supplementary Figure 2. **Optical microscope images of the three tDMG devices.** The twist angle of each device is denoted at the top left corner of each image. Devices are encapsulated in BN flakes with thicknesses of 10-30 nm. All scale bars are 10 μm.

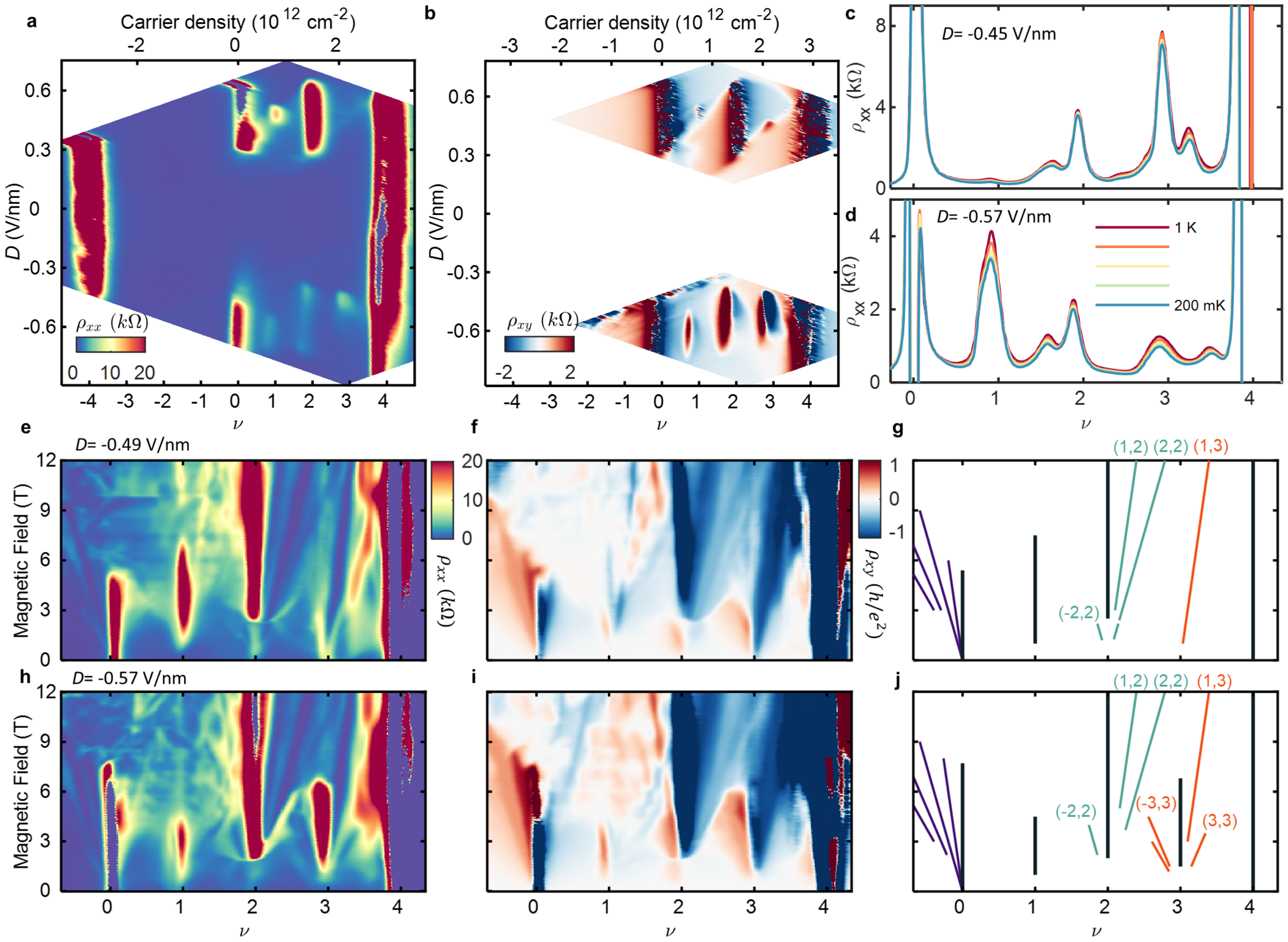

Supplementary Figure 3. **Additional transport characterization and Landau fan diagrams for $D < 0$ in device D1 ($\theta = 1.13°$).** Maps of **a**, ρ_{xx} at $B = 0$ and **b**, ρ_{xy} antisymmetrized with $|B| = 0.5$ T, acquired at $T = 300$ mK. The primary difference in ρ_{xx} at $B = 0$ compared with the symmetrized map at $|B| = 0.5$ T shown in Fig.1a of the main text is the complete absence of a correlated insulating state at $\nu = 3$. **c-d**, ρ_{xx} as a function of filling factor at $D = -0.45$ V/nm (**c**) and $D = -0.57$ (**d**), acquired at different T below 1 K. All correlated states at $\nu = 1$, 2, and 3 exhibit metallic temperature dependence. **e-j**, Landau fan diagrams at $D = -0.49$ V/nm (**e-g**) and $D = -0.57$ V/nm (**h-j**), acquired at $T = 300$ mK. The leftmost column shows ρ_{xx}, the central column shows ρ_{xy}, and the rightmost column schematically denotes the observed states following the convention established in Fig.3 of the main text. In **e-g**, we observe a very strong $(1, 3)$ state that emerges and becomes quantized at very small B. This likely reflects the anticipated $C_v = 1$ of the band at $D < 0$, however we are unable to determine this unambiguously in the absence of a QAH state at $B = 0$. We additionally observe a clear Landau fan corresponding to the correlated state at $\nu = 2$ at very low fields in (**e-g**). These gapped states close at the phase transition ($B \sim 2.8$ T) and immediately reopen at higher field along with a robust trivial insulating state, $(0, 2)$. Although this implies a first-order phase transition between two different correlated states, we are not able to unambiguously identify the ground state order of either from our measurements.

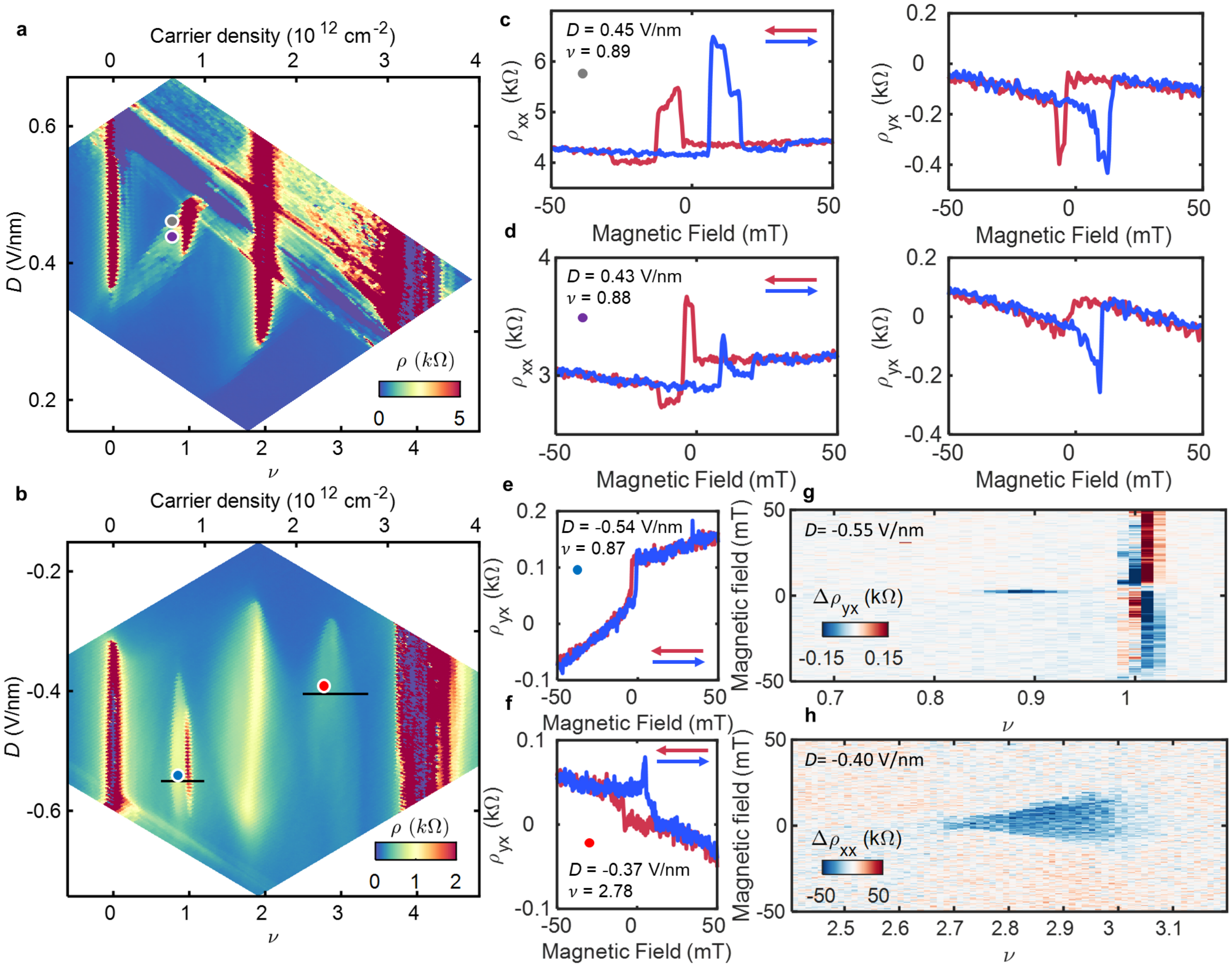

Supplementary Figure 4. **Additional transport characterization and AHE in device D2 ($\theta = 1.19°$).** Maps of **a**, ρ_{xx} at $B = 0$ and **b**, ρ_{xy} antisymmetrized with $|B| = 0.5$ T, acquired at $T = 15$ mK and $T = 500$ mK respectively. Artifacts due to poor contacts obscure a portion of the map in (**a**), preventing an analysis of the $\nu = 3$ state for $D > 0$ in this device. **c-d**, ρ_{xx} (top) and ρ_{yx} (bottom) acquired as B is swept back and forth at ν and D indicated by the labels and the associated gray and purple markers in (**a**), acquired at $T = 500$ mK. Similar to device D1 shown in Fig.1 of the main text, we observe AHE for $D > 0$ only within the "halo" region ($\nu < 1$) associated with the correlated insulating state at $\nu = 1$. No obvious AHE signatures are observed precisely at $\nu = 1$. **e-f**, ρ_{yx} acquired as B is swept back and forth at ν and D indicated by the labels and the associated blue and red markers in (**b**). We observe a weak AHE in the correlated metallic states nearby both $\nu = 1$ and 3, qualitatively similar to the behavior of device D1 as shown in Fig.1 of the main text. Data are acquired at $T = 500$ mK for **e** and at $T = 50$ mK for **f**. **g**, $\Delta\rho_{yx}$ as a function of doping at $D = -0.55$ V/nm. In contrast to all other devices, we observe a trivial insulating state precisely at $\nu = 1$, with no clear signatures of an AHE (see also Supplementary Information Figs. 8c-d). **h**, $\Delta\rho_{xx}$ as a function of doping at $D = -0.55$ V/nm. $\Delta\rho_{xx}$ is shown rather than $\Delta\rho_{yx}$ owing to mixing effects observed in the latter for the specific contact pairs tested. Data in **g-h** is acquired at $T = 300$ mK.

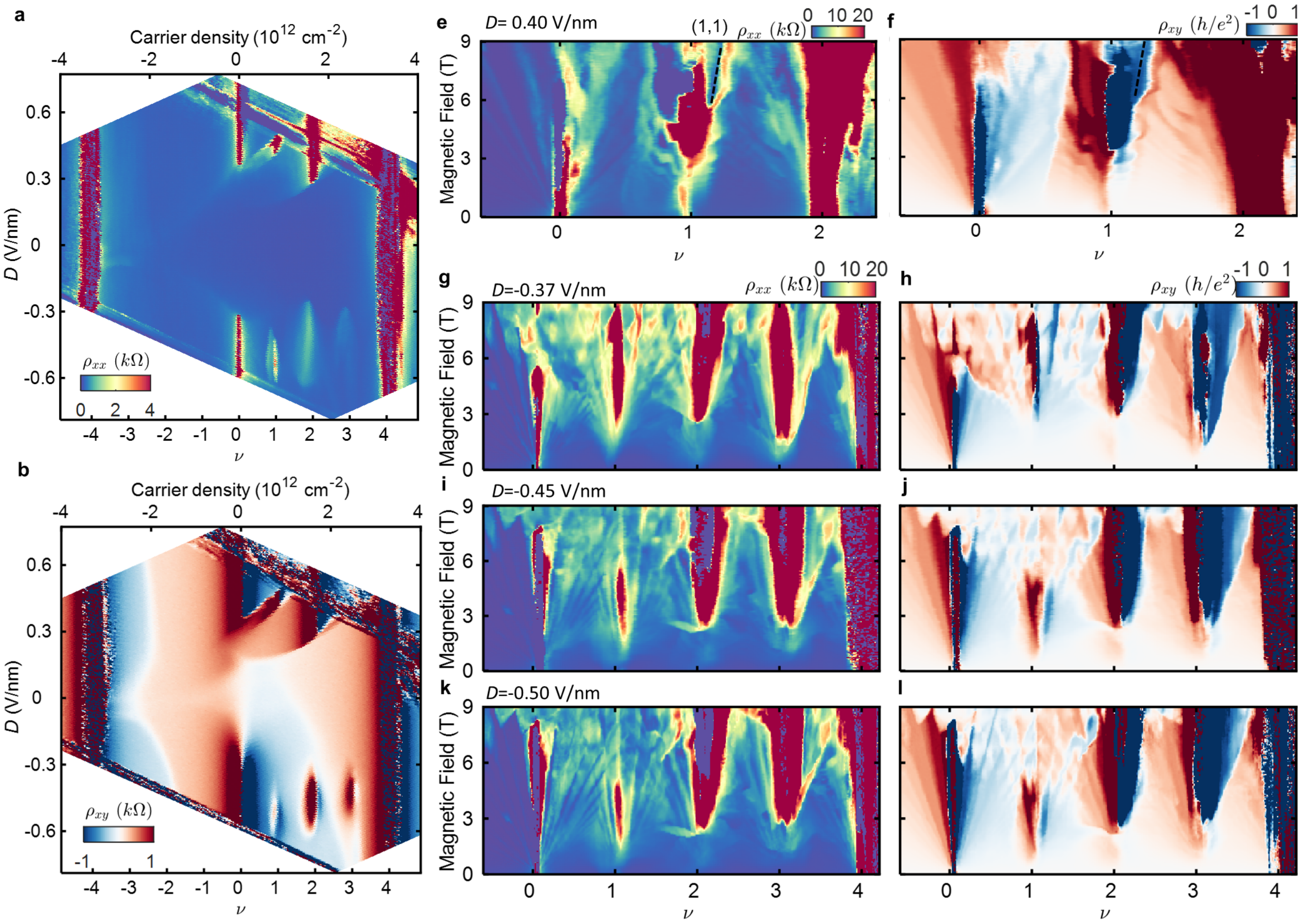

Supplementary Figure 5. **Additional transport characterization and Landau fan diagrams in device D2 ($\theta = 1.19°$).** Maps of **a**, ρ_{xx} at $B = 0$ and **b**, ρ_{xy} antisymmetrized with $|B| = 0.5$ T, acquired at $T = 15$ mK and $T = 500$ mK respectively. **c-d**, Landau fan diagram at $D = 0.40$ V/nm, acquired at $T = 100$ mK. Similar to device D1 shown in the main text, we observe a trivial insulating state at $\nu = 1$ for small B, which appears to cede to a $(1, 1)$ Chern insulator state at higher field (marked by dashed black line). States near $\nu = 3$ are obscured by poor contacts, and are not shown. **e-j**, Landau fan diagrams at $D = -0.37$ V/nm (**g-h**), $D = -0.45$ V/nm (**i-j**), and $D = -0.50$ V/nm (**k-l**), acquired at $T = 100$ mK. The left column shows ρ_{xx} and the right column shows ρ_{xy}. We find that the critical fields, B_c, corresponding to the onset of insulating states at $\nu = 1$, 2, and 3 depend on D. All exhibit signatures of first-order phase transitions at or near $\nu = 2$ and 3, but a continuous onset of insulating behavior at $\nu = 1$.

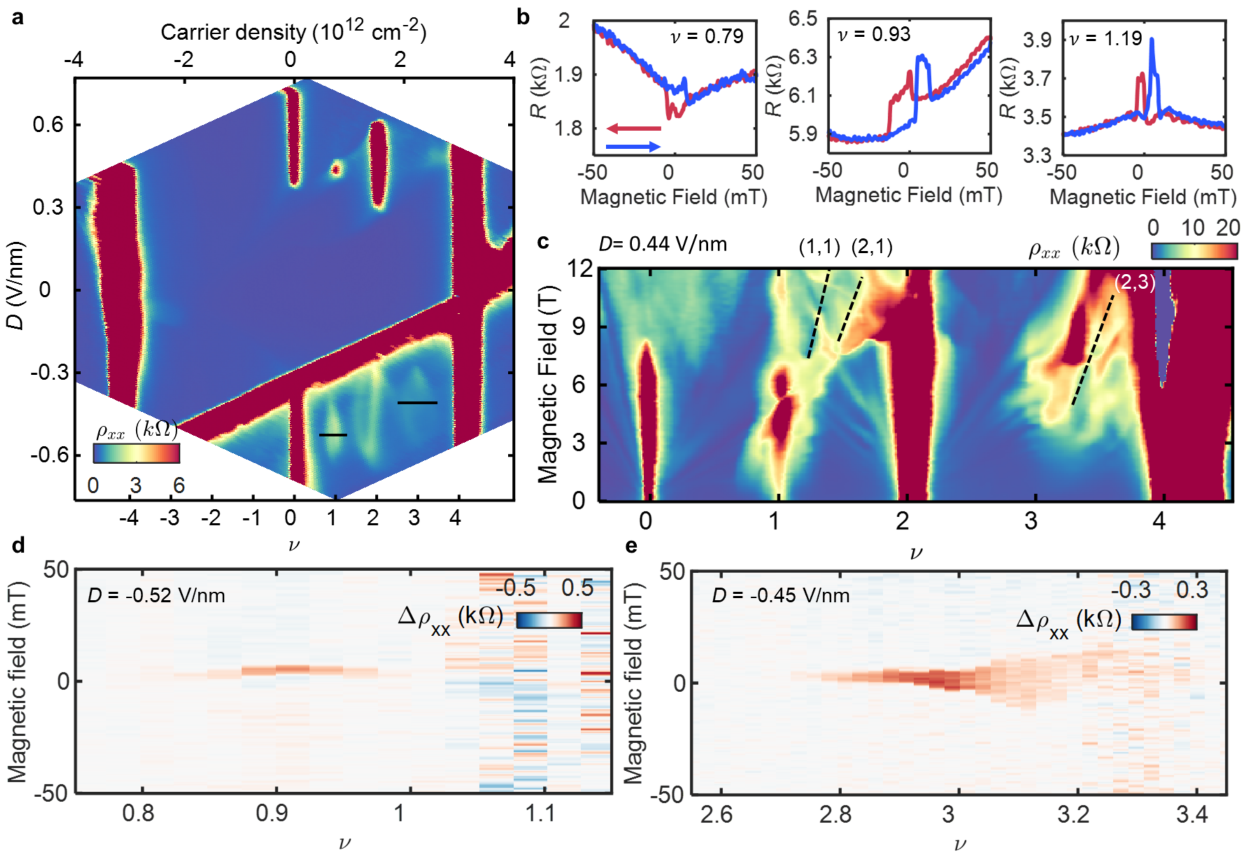

Supplementary Figure 6. **Transport characterization, AHE, and Landau fan diagrams in device D3 ($\theta = 1.14°$). a**, Map of ρ_{xx} at $B = 0$, acquired at $T = 15$ mK. This device did not have suitable working contacts to measure the corresponding ρ_{xy} without large mixing with ρ_{xx}. **b**, AHE shown at different ν near $\nu = 1$ with $D = 0.46$ V/nm. R is shown here due to lack of proper ρ_{yx} contacts, it thus exhibits features of both ρ_{xx} and ρ_{yx}. Similar to devices D1 and D2, we observe the AHE nearby $\nu = 1$, but trivial insulating behavior at $\nu = 1$. **c**, Landau fan diagram at $D = 0.44$ V/nm, acquired at $T = 300$ mK. Similar to devices D1 and D2, we observe a trivial insulating state at $\nu = 1$ for small B, which appears to cede to a sequence of correlated Chern insulator states at higher field. Chern insulator states corresponding to $\nu = 3$ emerge only at high field, similar to device D1. **d-e**, $\Delta\rho_{xx}$ as a function of doping at $D = -0.52$ V/nm nearby $\nu = 1$ (**d**) and $D = -0.45$ V/nm nearby $\nu = 3$ (**e**), acquired at $T = 300$ mK, as indicated by the black lines in **a**. We observe clear AHE for a wide range around $\nu = 3$, and signatures of a very weak AHE near $\nu \sim 0.9$.

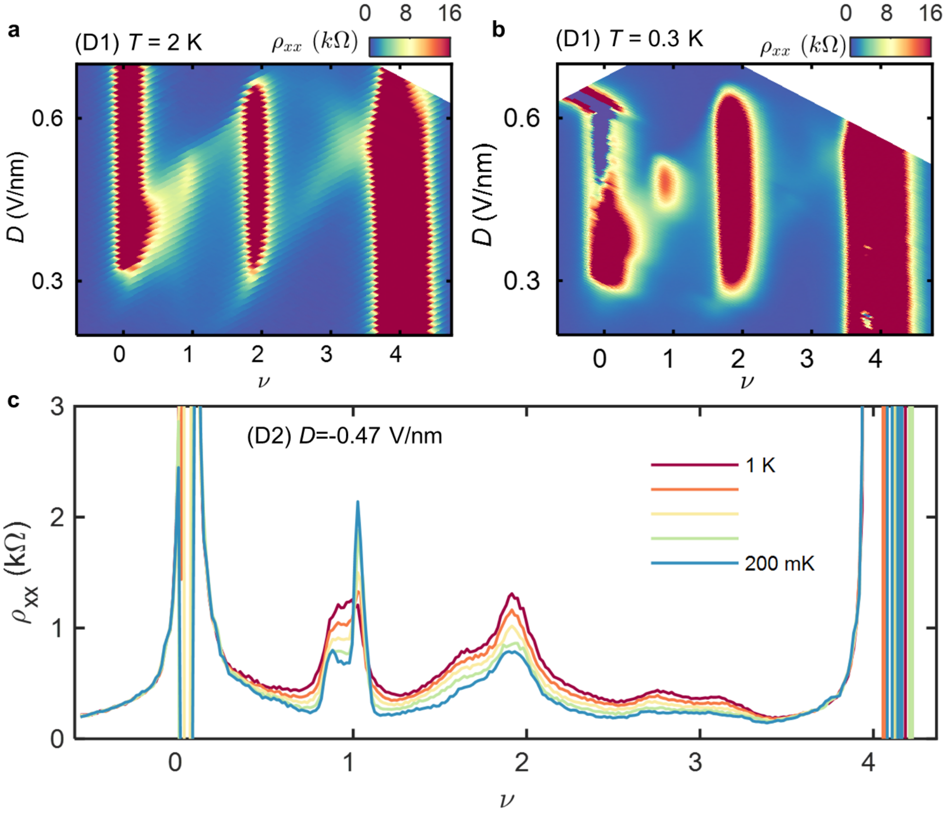

Supplementary Figure 7. **Temperature dependence of correlated insulating states at $\nu = 1$. a-b**, Maps of ρ_{xx} for $D > 0$ in device D1, acquired at $T = 2$ K (**a**) and $T = 0.3$ K (**b**). Over a small range of D, the resistivity at $\nu = 1$ increases with decreasing temperature, marking insulating behavior. **c**, ρ_{xx} as a function of filling factor at $D = -0.47$ V/nm in device D2, measured at selected temperatures from 1 K to 200 mK, in steps of 200 mK. A correlated insulating state emerges precisely at $\nu = 1$, as indicated by the sharp peak in ρ_{xx} emerging as the temperature is lowered. All other correlated states remain metallic down to base temperature.

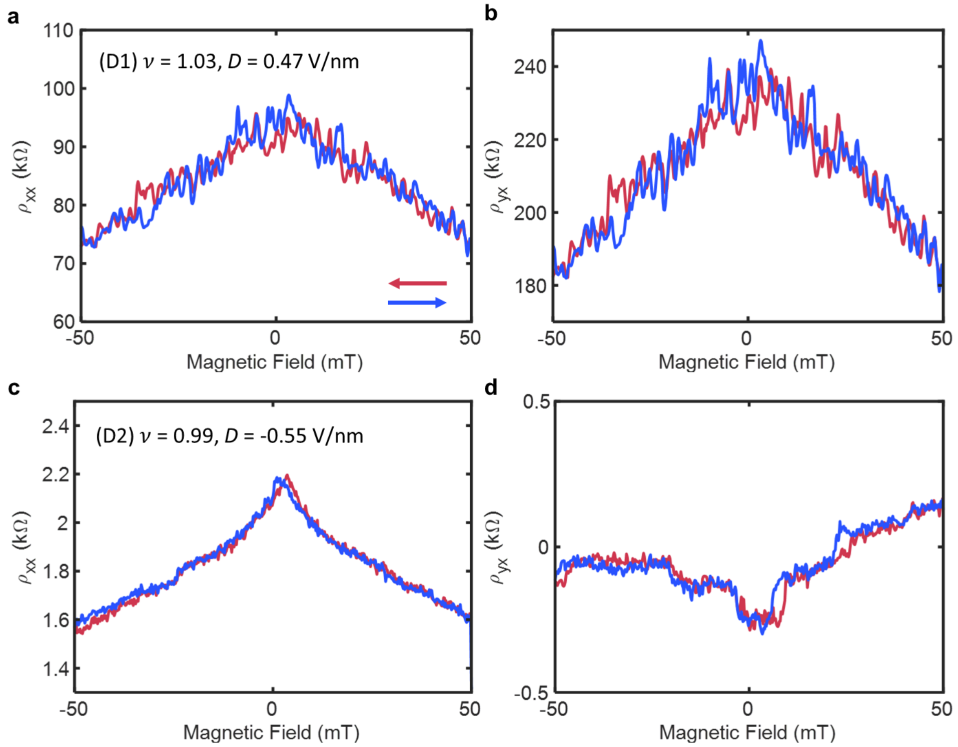

Supplementary Figure 8. **Absence of the AHE precisely at $\nu = 1$ for insulating states.** **a**, ρ_{xx} and **b**, ρ_{yx} measured as B is swept back and forth at $\nu = 1.03$ for $D = 0.47$ V/nm in device D1. **c**, ρ_{xx} and **d**, ρ_{yx} measured as B is swept back and forth at $\nu = 0.99$ for $D = -0.55$ V/nm in device D2. In both cases, insulating behavior is observed at $\nu = 1$ (see Supplementary Information Fig. 7). Neither exhibit any clear signatures of AHE; most notably, we do not observe a hysteresis loop encircling $B = 0$. The state at $\nu = 1$ has very high resistance in device D1, and the measured ρ_{yx} appears to have large mixing with ρ_{xx}. Owing to the noise in the measurements and the insulating behavior of the state, calculating $\Delta\rho_{yx}$ leads to rapidly oscillating negative and positive values, as seen in Fig.1e of the main text. We observe abrupt but small jumps at various values of $|B| > 0$ in (**d**). We do not know the origin of these features, and although they may potentially indicate some form of disordered magnetism, they are distinct from the single hysteresis loops around $B = 0$ observed for $\nu < 1$, as shown in Supplementary Information Figs. 4e and g. We also observe negative magnetoresistance in **a** and **c** that is consistent with the magnetic field-induced suppression of the insulating states detailed in Fig. 2 of the main text.

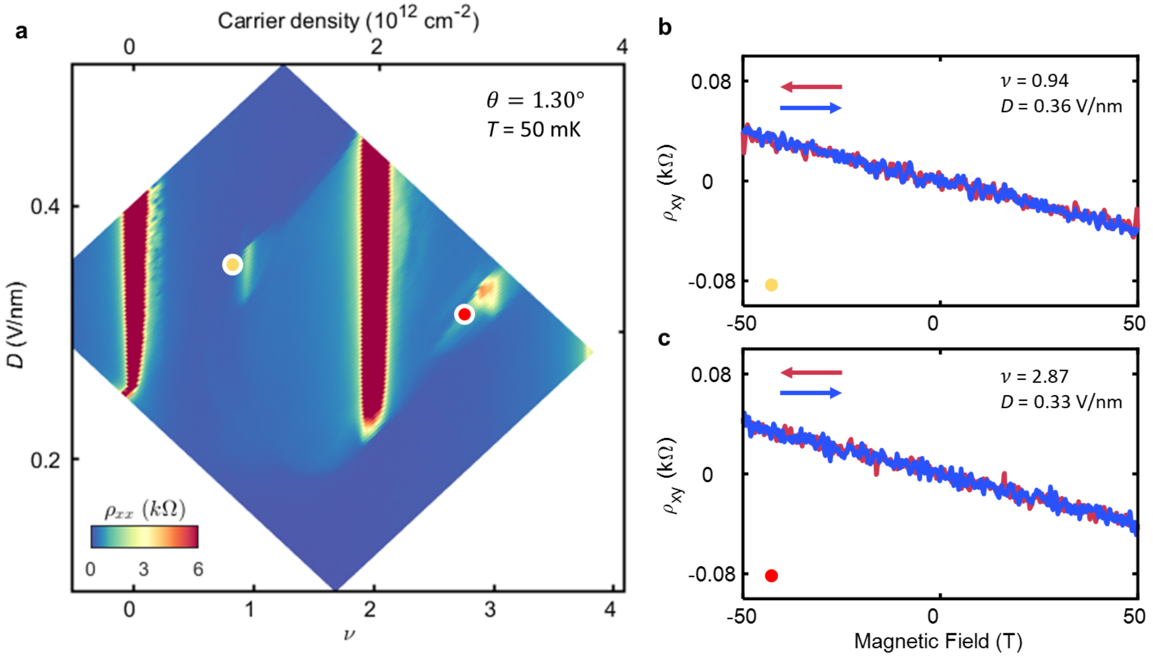

Supplementary Figure 9. **Absence of the AHE near $\nu = 1$ and 3 in tDBG.** **a**, Map of ρ_{xx} for a twisted double bilayer graphene (tDBG) device with $\theta = 1.30°$. We observe a robust correlated insulating state at $\nu = 2$, and very weakly developed correlated insulating states at $\nu = 1$ and 3. We also observe small "halo" features surrounding $\nu = 1$ and 3, indicating the formation of new symmetry-broken Fermi surfaces at each. **b-c**, Antisymmetrized ρ_{xy} measured as the field is swept back and forth near $\nu = 1$ (**b**) and $\nu = 3$ (**c**), at ν and D indicated by the labels and the associated yellow and red markers in (**a**). We do not observe any signatures of an AHE or hysteresis in these measurements, nor at any other measured value of ν and D.

REFERENCES

[1] Bultinck, N. *et al.* Ground state and hidden symmetry of magic-angle graphene at even integer filling. *Physical Review X* **10**, 031034 (2020).

[2] Chen, S. *et al.* Electrically tunable correlated and topological states in twisted monolayer–bilayer graphene. *Nature Physics* **17**, 374–380 (2021).

[3] Polshyn, H. *et al.* Electrical switching of magnetic order in an orbital chern insulator. *Nature* **588**, 66–70 (2020).

[4] Xu, S. *et al.* Tunable van hove singularities and correlated states in twisted monolayer–bilayer graphene. *Nature Physics* 1–8 (2021).

[5] Park, Y., Chittari, B. L. & Jung, J. Gate-tunable topological flat bands in twisted monolayer-bilayer graphene. *Physical Review B* **102**, 035411 (2020).
